# Prognostic Value of Tumor-Infiltrating FoxP3^+^ T Cells in Gastrointestinal Cancers: A Meta Analysis

**DOI:** 10.1371/journal.pone.0094376

**Published:** 2014-05-14

**Authors:** Yong Huang, Huaiwei Liao, Yong Zhang, Rongfa Yuan, Fengmei Wang, Yingtang Gao, Peng Wang, Zhi Du

**Affiliations:** 1 Tianjin Medical University, Tianjin, China; 2 Department of Hepatobiliary Surgery, The Second Affiliated Hospital of Nanchang University, Nanchang, China; 3 Department of plastic surgery, The First Affiliated Hospital of Nanchang University, Nanchang, China; 4 Department of Gastroenterology and Hepatology, The Third Central Hospital of Tianjin, Tianjin, China; 5 Key Laboratory of Artificial Cell, Institute of Hepatobiliary Disease, The Third Central Hospital of Tianjin, Tianjin, China; Okayama University, Japan

## Abstract

**Purpose:**

Tumor-infiltrating FoxP3^+^ T cells have been reported in various human tumors, which impaired cell-mediated immunity and promoted disease progression. However, its prognostic value for survival in patients with different gastrointestinal cancers [hepatocellular carcinoma (HCC), colorectal cancer (CRC), gastric cancer (GC)] remains controversial.

**Methods:**

Relevant literature was searched using PubMed, Embase, Cochrane, Ovid Medline and Chinese wanfang databases. A meta-analysis was conducted to estimate pooled survival and recurrence ratios. The odds ratio (OR) and 95% confidence intervals (CI) were calculated employing fixed- or random-effects models depending on the heterogeneity of the included trials.

**Results:**

For HCC and GC, the overall survival at 1, 3 and 5-year of high FoxP3^+^ T cells infiltration patients were lower than low FoxP3^+^ T cells infiltration patients (*P*<0.05). The recurrences at 1, 3 and 5-year of high FoxP3^+^ T cells infiltration patients were higher than low FoxP3^+^ T cells infiltration patients (*P*<0.001). But for CRC, the overall survival at 1, 3 and 5-year of high FoxP3^+^ T cells infiltration patients were higher than low FoxP3^+^ T cells infiltration patients (*P*<0.001). There were no differences in 1, 3 and 5-year recurrences between high and low FoxP3^+^ T cells infiltration patients (*P*>0.05).

**Conclusions:**

Our findings suggested that tumor-infiltrating FoxP3^+^ T cells were a factor for a poor prognosis for HCC and GC, but a good prognosis for CRC.

## Introduction

Immune cells that infiltrate tumors engage in an extensive and dynamic crosstalk with cancer cells and some of the molecular events that mediate this dialog have been revealed [1]. In the past decade, much effort has been devoted to finding the function of regulatory T cells (Tregs) in tumor. Tregs are a subgroup of CD4^+^ T helper cells with the function of suppressing T-cell immunity in both physiologic and disease statuses. Forkhead box protein P3 (FoxP3) is a transcription factor necessary and sufficient for induction of the immunosuppressive functions of Tregs, and it is now considered as the most specific marker for Tregs in tumors [2].

Abundance tumor-infiltrating FoxP3^+^ T cells are expected to be associated with an unfavorable prognosis, as expected from their capacity to inhibit antitumor immunity. However, this idea has been challenged by recent studies showing that, high tumor infiltration by FoxP3^+^ T cells is not always associated with a poor prognosis. On the contrary, it can improve survival in some tumors [3]–[5]. It was inconsistent with the initial hypothesis that FoxP3^+^ T cells inhibit antitumor immunity. Even in the same kind of tumor, this conclusion was not entirely consistent [4], [5]. The discrepancy was very obvious, especially in the gastrointestinal cancers such as hepatocellular carcinoma (HCC), colorectal cancer (CRC) and gastric cancer (GC) which all were considered as inflammation-associated cancers since with rich exogenous antigens.

To investigate this apparent discrepancy, we sought to conduct a meta-analysis to estimate the prognostic importance of tumor-infiltrating FoxP3^+^ T cells level for overall survival (OS) and disease-free survival (DFS) among patients with HCC, CRC and GC, aiming to gain insights into whether FoxP3^+^ T cells could provide useful guidance in the biological understanding and treatment of solid tumors.

## Materials and Methods

### Literature search

Relevant articles were identified by two reviewers via an electronic search of PubMed, EMBASE, Cochrane, Ovid Medline and Chinese wanfang databases using the following keywords: (FoxP3 or regulatory T cells), (hepatocellular carcinoma, colorectal cancer or gastric cancer) and “prognosis”. And the search time period of the electronic database was from inception to Feb 8th, 2014. Additionally, possible missing papers were searched in reference lists of selected papers and systematic review. A search for unpublished literature was not performed. Disagreement on article inclusion between the two reviewers was resolved via a third reviewer.

### Inclusion and exclusion criteria

Inclusion criteria for this study were as follows: (1) patients were diagnosed clearly; (2) report of FoxP3^+^ T cells in tumor surgical specimens; (3) FoxP3^+^ T cells evaluation using immunohistochemical method; (4) association of high and low FoxP3^+^ T cells infiltration patients with overall survival (OS), and/or disease-free survival (DFS) and contained survival curves. (5) when the same author or group reported results obtained from the same patient population in more than one article, the most recent report or the most informative report was included.

Exclusion criteria for this study were as follows: (1) letters, reviews, case reports, conference abstracts, editorials, and expert opinion were excluded; (2) articles in which have no information on survival rates or survival curve; (3) Non-surgical treatment study; (4) non-primary cancer, such as metastatic cancer or recurrent cancer; (5) peripheral blood or peritumoral specimens.

Name of authors or journals of the articles did not influence our decision in excluding or including the articles.

### Statistical analysis

Hazard ratio (HR) and its 95% confidence interval (CI) were used to estimate the association between FoxP3^+^ T cells and patients' prognosis. If a direct report of survival and recurrence ratios were not available, then the survival data read from Kaplan-Meier curves were read by Engauge Digitizer version 4.1 (http://digitizer.sourceforge.net/) as described previously [6]–[8]. This work was performed by two independent persons to reduce inaccuracy in the extracted survival rates.

All analyses were performed with Review Manager version 5 (RevMan, Cochrane Collaboration, Oxford, England). Statistical heterogeneity between trials was evaluated by χ^2^ test and was considered significant when *P*<0.05. In the absence of statistically significant heterogeneity, the Mantel-Haenszel method in the fixed-effect model was used for the Meta analysis. Otherwise, the DerSimonian and Laird method in the random-effect model was selected. The odds ratio (OR) with 95%CI was used to assess treatment efficacy. The combined result was an average OR and 95%CI weighted according to the standard error of the OR of the trial. *P*<0.05 was considered statistically significant. We used funnel plots to assess the publication bias, and tested for funnel plot asymmetry using Egger's test and Begg's test.

## Results

### Study selection and characteristics

**Figure 1:**
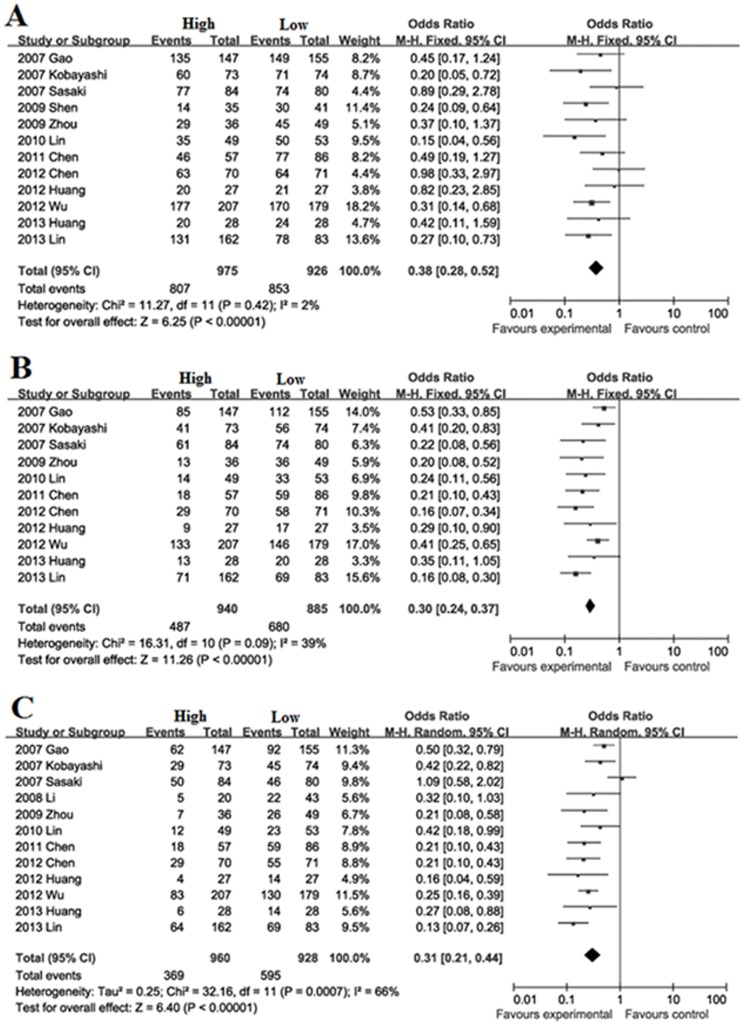
Forest plot of Hazard ratio (HR) for survival of HCC patients. Fixed effect model of odds ratio for survival of follow-up 1(A), 3-year (B) and random effect model of odds ratio for survival of follow-up 5-year (C) of HCC patients after surgery: high FoxP3^+^ T cells infiltration patients *vs* low FoxP3^+^ T cells infiltration patients.

For HCC, 13 eligible trials involving 1964 patients were ultimately identified in Table 1 [9]–[21]. For CRC, 10 eligible trials involving 2756 patients were ultimately identified in Table 2 [22]–[31]. For GC, 16 eligible trials involving 1873 patients were ultimately identified Table 3 [32]–[47]. FigureS1A (for HCC), FigureS1B (for CRC) and FigureS1C (for GC) illustrate the search process and the final selection of relevant studies.

**Table 1:** Main characteristics of studies about HCC included in the meta-analysis.

Author	Year	Journal	Quality score	Number of cases		Marker	Antibody	Cutoff	Survival
				M/F	High/low				
Gao[9]	2007	J Clin Oncol	6	260/42	147/155	FoxP3	Biolegend	Median	OS,DFS
Kobayashi[10]	2007	Clin Cancer Res	6	113/34	73/74	FoxP3/CD4	Novocastra	Median	OS,DFS
Sasaki[11]	2007	Eur J Surg Oncol	6	126/38	84/80	FoxP3	Abcan	Median	OS,DFS
Li[12]	2008	Zhonghua Zhong Liu	7	54/9	20/43	FoxP3	Abcan	Other	OS
Shen[13]	2009	Can J Surg	6	70/6	35/41	FoxP3	Abcan	Median	OS,DFS
Zhou[14]	2009	Int J Cancer	7	-	36/49	FoxP3	Abcan	Median	OS,DFS
Lin[15]	2010	Chin J Cancer	6	85/17	49/53	FoxP3	Abcan	Median	OS
Chen[16]	2011	PLoS One	7	-	57/86	FoxP3	Abcan	Median	OS,DFS
Chen[17]	2011	Med Oncol	7	-	70/71	FoxP3	Abcan	Median	OS,DFS
Huang[18]	2012	Digestion	7	45/9	27/27	FoxP3	Abcan	Median	OS,DFS
Wu[19]	2012	J Gastroenterol Hepatol	7	341/45	207/179	FoxP3	Abcan	Other	OS,DFS
Huang[20]	2013	J Gastroenterol Hepatol	7	50/6	28/28	FoxP3	Abcan	Median	OS,DFS
Lin[21]	2013	Cancer Prev Res	7	-	162/83	FoxP3/CD4	Abcan	Other	OS,DFS

F, female; M, male; Quality score was assessed using the validated Jadad scale; High, high FoxP3^+^ T cells infiltration; Low, low FoxP3^+^ T cells infiltration.

**Table 2:** Main characteristics of studies about CRC included in the meta-analysis.

Author	Year	Journal	Quality score	Number of cases		Marker	Antibody	Cutoff	Survival
				M/F	High/low				
Sinicrope[22]	2009	Gastroenterology	7	84/76	101/59	FoxP3	Abcan	Other	DFS
Lee[23]	2010	Cancer	7	29/34	39/24	FoxP3	eBioscience	Other	OS
Suzuki[24]	2010	Cancer Immunol Immunother	6	53/41	30/64	FoxP3	Abcan	Mean	OS,DFS
Frey[25]	2010	Int J Cancer	6	-	614/616	FoxP3	Abcan	Other	OS,DFS
Nosho[26]	2010	J Pathol	6	-	384/384	FoxP3	BioLegend	Other	OS
Tosolini[27]	2011	Cancer Research	5	-	18/38	FoxP3	Abcan	Other	DFS
Yoon[28]	2012	PLoS One	7	-	78/78	FoxP3	Abcan	Median	OS
Suzuki[29]	2013	Clinical Immunology	6	49/39	34/54	FoxP3	Abcan	Mean	OS.DFS
Zeestraten[30]	2013	Cancer Microenvironment	6	44/36	38/38	FoxP3	Abcan	Median	OS,DFS
Kim[31]	2013	PLoS One	7	37/28	27/38	FoxP3	Abcan	Mean	OS

F, female; M, male; Quality score was assessed using the validated Jadad scale; high FoxP3^+^ T cells infiltration; Low, low FoxP3^+^ T cells infiltration.

**Table 3:** Main characteristics of studies about GC included in the meta-analysis.

Author	Year	Journal	Quality score	Number of cases		Marker	Antibody	Cutoff	Survival
				M/F	High/low				
Mizukami[32]	2008	Br J Cancer	7	56/24	40/40	FoxP3	eBioscience	Median	OS
Perrone[33]	2008	Eur J Cancer	7	53/57	58/52	FoxP3	eBioscience	Median	OS,DFS
Haas[34]	2009	BMC Gastroenterol	6	40/12	26/26	FoxP3	Abcan	Median	OS
Shen[35]	2010	J Cancer Res Clin Oncol	7	89/44	66/67	FoxP3	Biolegend	Median	OS
Du[36]	2011	Cancer Sci	6	131/48	87/92	FoxP3	Abcam	Median	OS,DFS
Kim[37]	2011	J Surg Oncol	7	126/54	90/90	FoxP3/CD4	Abcan	Median	OS,DFS
Lu[38]	2011	J Surg Oncol	7	-	30/30	FoxP3	Abcan	Median	OS
Shu[39]	2011	Zhonghua weichangwaike	7	-	45/43	FoxP3	eBioscience	Median	OS
Wang[40]	2011	Ann Surg Oncol	7	69/38	53/54	FoxP3	Abcan	Median	OS
Ishigami[41]	2012	Cancer Immunol Immunother	7	99/42	76/65	FoxP3	Dako	Mean	OS
Kashimura[42]	2012	Gastric Cancer	6	89/34	62/61	FoxP3	Abcan	Median	OS,DFS
Yoshii[43]	2012	Br J Cancer	7	44/48	49/43	FoxP3	Abcan	Median	OS
Deng[44]	2013	PLoS One	6	70/29	48/51	FoxP3	Abcan	Median	OS
Kim[45]	2013	Hum Pathol	6	55/44	49/50	FoxP3	Abcan	Median	OS
Zhou[46]	2013	PLoS One	7	89/44	87/46	FoxP3	Biolegend	Mean	OS
Ma[47]	2014	Br J Cancer	7	132/65	24/173	FoxP3	Abcam	Other	OS

F, female; M, male; Quality score was assessed using the validated Jadad scale; high FoxP3^+^ T cells infiltration; Low, low FoxP3^+^ T cells infiltration.

### Meta-analysis for HCC

Survival during follow-up 1, 3, 5-year after surgical resection: The overall survival rate during follow-up 1-year was significantly lower in high FoxP3^+^ T cells infiltration patients (82.8%) than low FoxP3^+^ T cells infiltration patients (92.1%) with a combined OR of 0.38 (95%CI = 0.28–0.52, *P*<0.001. Figure 1A). The overall survival rate during follow-up 3-year was significantly lower in high FoxP3^+^ T cells infiltration patients (51.8%) than low FoxP3^+^ T cells infiltration patients (76.8%) with a combined OR of 0.30 (95%CI = 0.24–0.37, *P*<0.001. Figure 1B). The overall survival rate during follow-up 5-year was significantly lower in high FoxP3^+^ T cells infiltration patients (38.4%) than low FoxP3^+^ T cells infiltration patients (64.1%) with a combined OR of 0.31 (95%CI = 0.21–0.44, *P*<0.001. Figure 1C).

**Figure 2:**
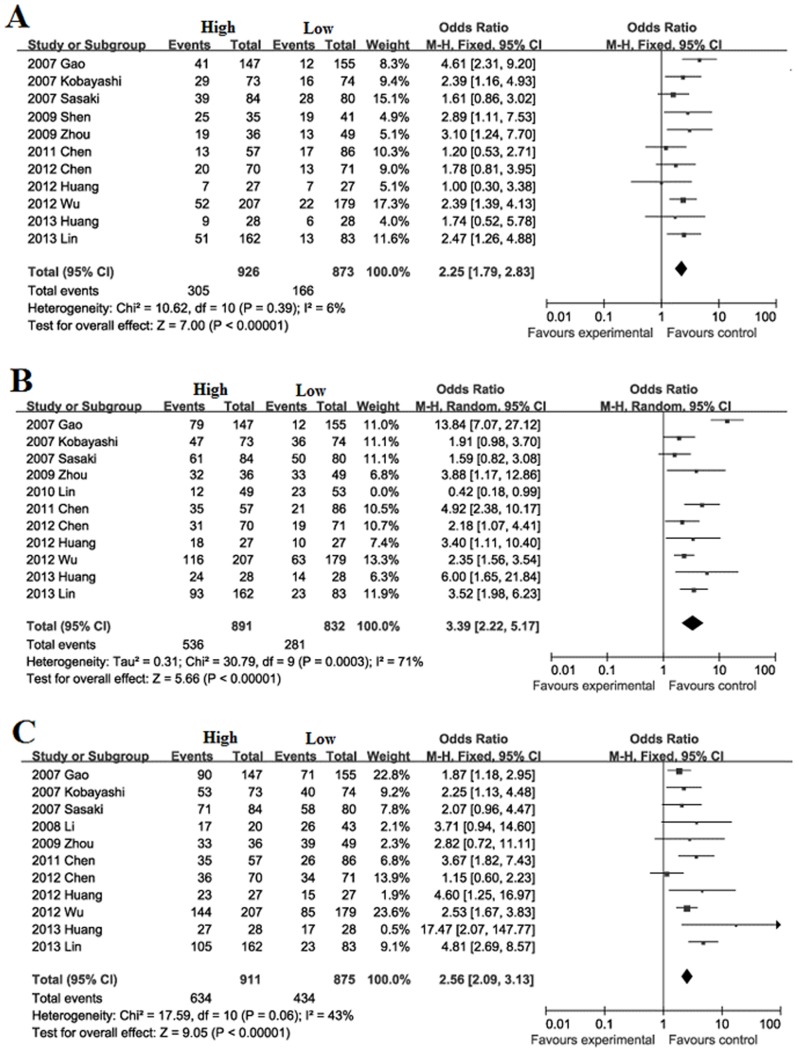
Forest plot of HR for recurrence of HCC patients. Fixed effect model of odds ratio for recurrence of follow-up 1(A), 5-year (C) and random effect model of odds ratio for recurrence of follow-up 3-year (B) of HCC patients after surgery: high FoxP3^+^ T cells infiltration patients *vs* low FoxP3^+^ T cells infiltration patients.

Recurrence during follow-up 1, 3, 5-year after surgical resection: The recurrence rate during follow-up 1-year was significantly higher in high FoxP3^+^ T cells infiltration patients (32.9%) than low FoxP3^+^ T cells infiltration patients (19.0%) with a combined OR of 2.25 (95%CI = 1.79–2.83, *P*<0.001. Figure 2A). The recurrence rate during follow-up 3-year was significantly higher in high FoxP3^+^ T cells infiltration patients (60.2%) than low FoxP3^+^ T cells infiltration patients (33.8%) with a combined OR of 3.39 (95%CI = 2.22–5.17, *P*<0.001. Figure 2B). The recurrence rate during follow-up 5-year was significantly higher in high FoxP3^+^ T cells infiltration patients (69.6%) than low FoxP3^+^ T cells infiltration patients (49.6%) with a combined OR of 2.56 (95%CI = 2.09–3.13, *P*<0.001. Figure 2C).

### Meta-analysis for CRC

**Figure 3:**
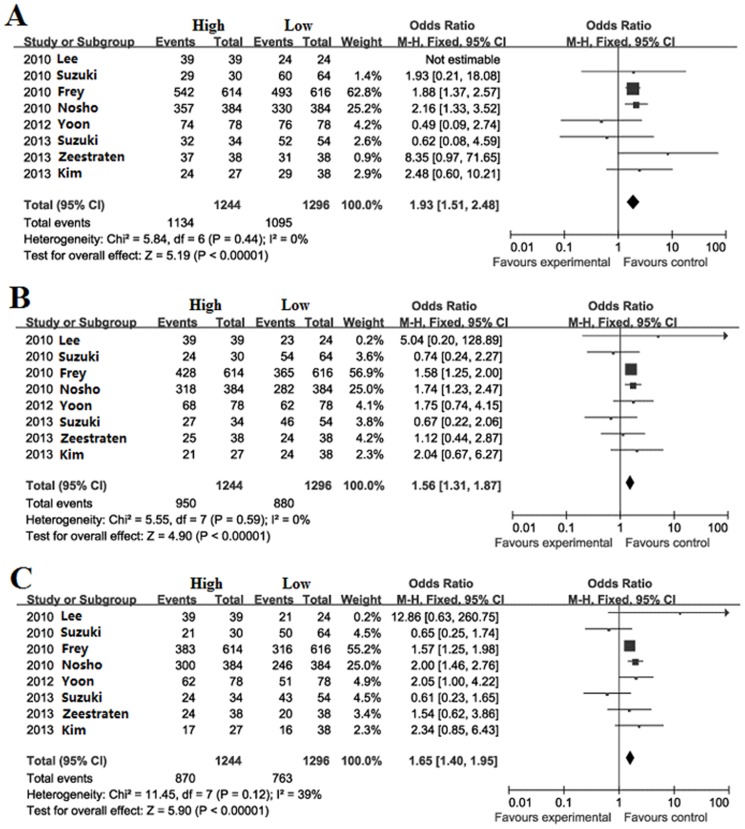
Forest plot of HR for survival of CRC patients. Fixed effect model of odds ratio for survival of follow-up 1 (A), 3 (B), 5-year (C) of CRC patients after surgery: high FoxP3^+^ T cells infiltration patients *vs* low FoxP3^+^ T cells infiltration patients.

Survival during follow-up 1, 3, 5-year after surgical resection: The overall survival rate during follow-up 1-year was significantly higher in high FoxP3^+^ T cells infiltration patients (91.2%) than low FoxP3^+^ T cells infiltration patients (84.5%) with a combined OR of 1.93 (95%CI = 1.51–2.48, *P*<0.001. Figure 3A). The overall survival rate during follow-up 3-year was significantly higher in high FoxP3^+^ T cells infiltration patients (76.4%) than low FoxP3^+^ T cells infiltration patients (67.9%) with a combined OR of 1.56 (95%CI = 1.31–1.87, *P*<0.001. Figure 3B). The overall survival rate during follow-up 5-year was significantly higher in high FoxP3^+^ T cells infiltration patients (69.9%) than low FoxP3^+^ T cells infiltration patients (58.9%) with a combined OR of 1.65 (95%CI = 1.40–1.95, *P*<0.001. Figure 3C).

**Figure 4:**
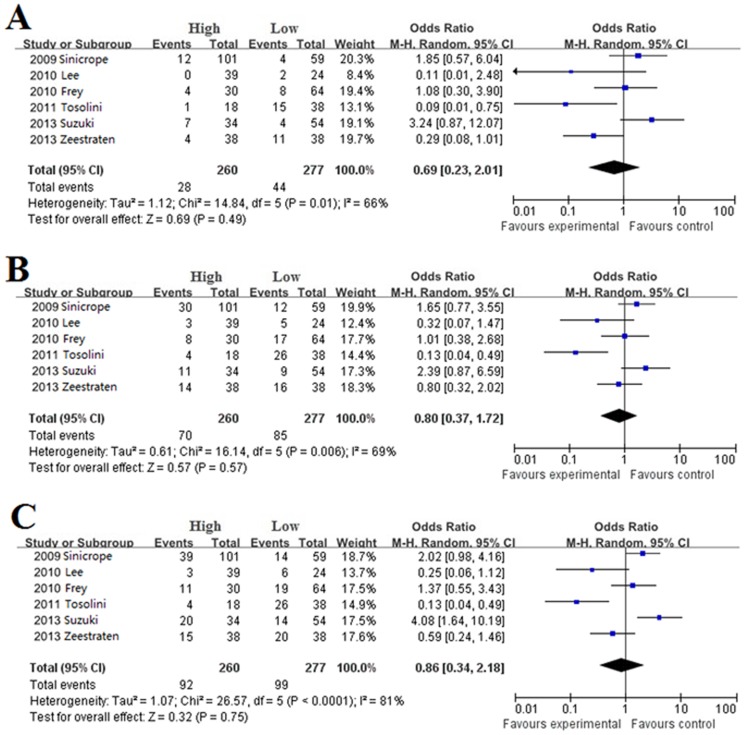
Forest plot of HR for recurrence of CRC patients. Random effect model of odds ratio for recurrence of follow-up 1 (A), 3 (B), 5-year (C) of CRC patients after surgery: high FoxP3^+^ T cells infiltration patients *vs* low FoxP3^+^ T cells infiltration patients.

**Figure 5:**
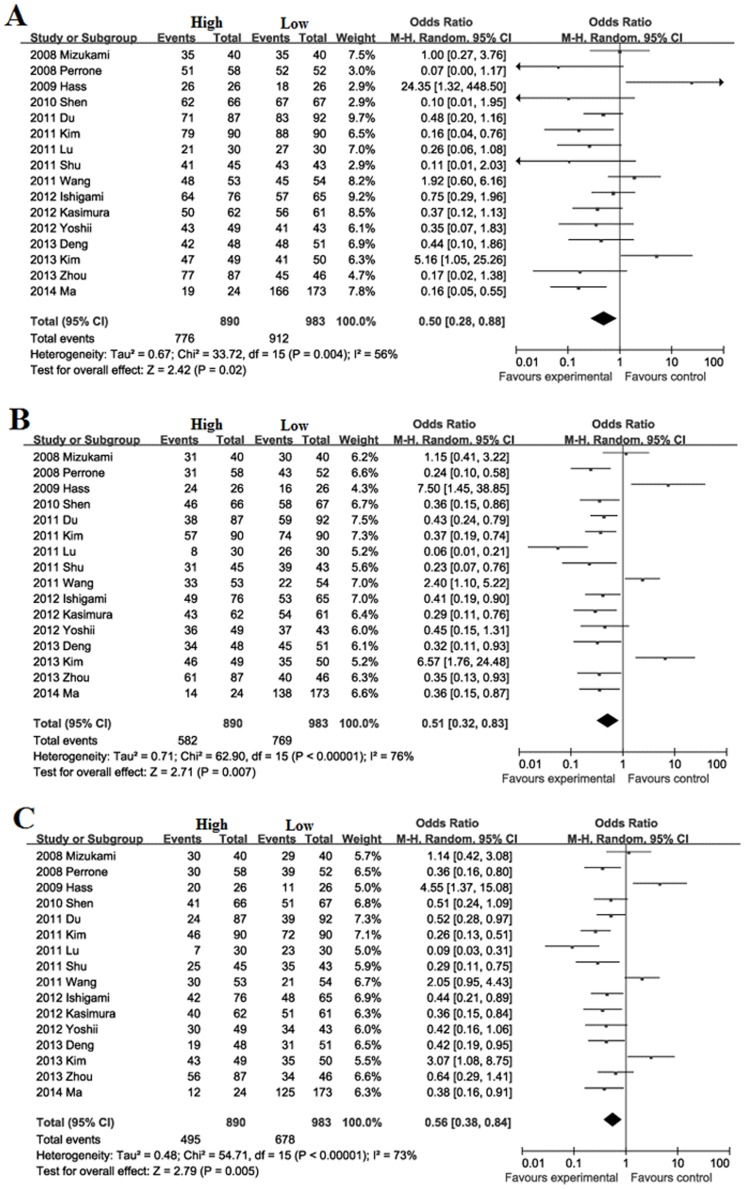
Forest plot of HR for survival of GC patients. Random effect model of odds ratio for survival of follow-up 1 (A), 3 (B), 5-year (C) of GC patients after surgery: high FoxP3^+^ T cells infiltration patients *vs* low FoxP3^+^ T cells infiltration patients.

Recurrence during follow-up 1, 3, 5-year after surgical resection: There were no differences in 1(OR = 0.69, 95%CI = 0.23–2.01, *P* = 0.49. Figure 4A), 3 (OR = 0.80, 95%CI = 0.37–1.72, *P* = 0.57. Figure 4B) and 5-year (OR = 0.86, 95%CI = 0.34–2.18, *P* = 0.75. Figure 4C) recurrences between high and low FoxP3^+^ T cells infiltration patients.

### Meta-analysis for GC

Survival during follow-up 1, 3, 5-year after surgical resection: The overall survival rate during follow-up 1-year was significantly lower in high FoxP3^+^ T cells infiltration patients (87.2%) than low FoxP3^+^ T cells infiltration patients (92.8%) with a combined OR of 0.50 (95%CI = 0.28–0.88, *P* = 0.02. Figure 5A). The overall survival rate during follow-up 3-year was significantly lower in high FoxP3^+^ T cells infiltration patients (65.4%) than low FoxP3^+^ T cells infiltration patients (78.2%) with a combined OR of 0.51 (95%CI = 0.32–0.83, *P* = 0.007. Figure 5B). The overall survival rate during follow-up 5-year was significantly lower in high FoxP3^+^ T cells infiltration patients (55.6%) than low FoxP3^+^ T cells infiltration patients (69.0%) with a combined OR of 0.56 (95%CI = 0.38–0.84, *P* = 0.005. Figure 5C).

**Figure 6:**
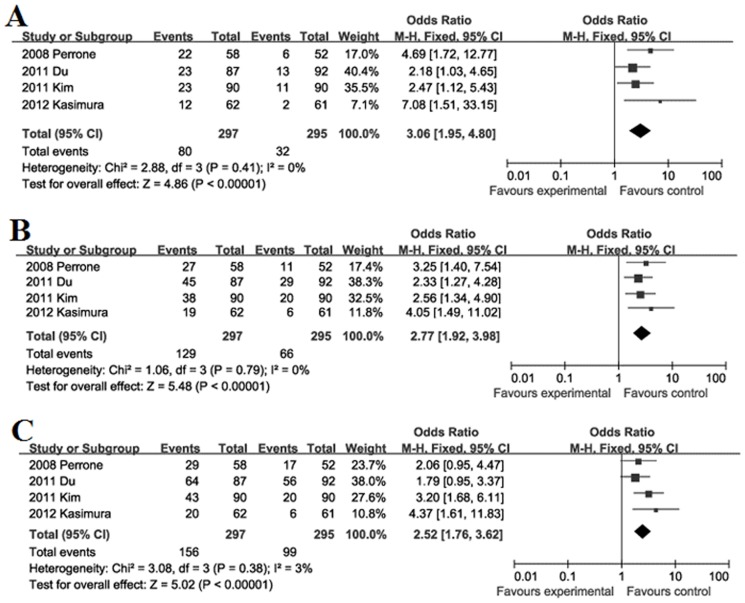
Forest plot of HR for recurrence of GC patients. Fixed effect model of odds ratio for recurrence of follow-up 1 (A), 3 (B), 5-year (C) of GC patients after surgery: high FoxP3^+^ T cells infiltration patients *vs* low FoxP3^+^ T cells infiltration patients.

Recurrence during follow-up 1, 3, 5-year after surgical resection: The recurrence rate during follow-up 1-year was significantly higher in high FoxP3^+^ T cells infiltration patients (26.9%) than low FoxP3^+^ T cells infiltration patients (10.8%) with a combined OR of 3.06 (95%CI = 1.95–4.80, *P*<0.001. Figure 6A). The recurrence rate during follow-up 3-year was significantly higher in high FoxP3^+^ T cells infiltration patients (43.4%) than low FoxP3^+^ T cells infiltration patients (22.4%) with a combined OR of 2.77 (95%CI = 1.92–3.98, *P*<0.001. Figure 6B). The recurrence rate during follow-up 5-year was significantly higher in high FoxP3^+^ T cells infiltration patients (52.5%) than low FoxP3^+^ T cells infiltration patients (33.6%) with a combined OR of 2.52 (95%CI = 1.76–3.62, *P*<0.001. Figure 6C).

### Publication bias

Publication bias may exist when no significant findings remain unpublished, thus artificially inflating the apparent magnitude of an effect. Survival and recurrences following high and low FoxP3^+^ T cells infiltration patients with HCC, CRC and GC were calculated by the fixed-effect model and random-effect model, respectively. The results were similar and the combined results were highly reliable.

The funnel plots on survival and recurrence following high and low FoxP3^+^ T cells infiltration patients with HCC (Figure S2), CRC (Figure S3) and GC (Figure S4) showed basic symmetry, which suggested no publication bias.

## Discussion

Tregs are functionally immunosuppressive subsets of CD4^+^ T, which were found by Sakaguchi et al [2] in 1995. They control the balance between tolerance and rejection of self and altered self by secreting IL-4, IL-10 and TGF-β and other cytokines [48]. For the identification of Tregs, many markers such as CTLA-4, GITR, OX-4, CD127 and transcription factor FoxP3 can be used [49]. FoxP3 is now considered as the most specific marker for Tregs [50], because it is critical for the development and function of Tregs. And FoxP3 became a popular single marker for Tregs studies in tumor. However, the conclusions from published research regarding its prognostic value for different tumors were controversial in different gastrointestinal cancers. Even in the same kind of tumor, this conclusion was not entirely consistent such as CRC and GC [3]–[5].

Meta-analysis is useful to integrate results from independent studies for a specified outcome. Pooled results from the combining relevant studies are statistical powerful, and make it possible to detecting effects that may be missed by individual studies. To date, no meta-analysis has been undertaken for any studies that evaluate tumor-infiltrating FoxP3^+^ T cells as a prognostic marker in HCC, CRC or GC. In this meta-analysis, 13 studies involving 1964 patients were analyzed. All the studies consistently shown high density of tumor-infiltrating FoxP3^+^ T cells have been associated with poor survival and high recurrences in HCC, consistent with the initial hypothesis that FoxP3^+^ T cells inhibit antitumor immunity. These conclusions were confirmed by our previous reports [18], [20], [51]. In all our data clarified the results of individual studies and to identify patients at high risk for whom specific- or adjuvant-therapy might be necessary since high density of FoxP3^+^ T cells is a prognostic factor for HCC.

For CRC, 10 studies involving 2756 patients were analyzed. Be different from HCC, studies of the prognostic value of FoxP3^+^ T cells in CRC have lead to highly discrepant findings. Some studies investigating colorectal cancer concluded that FoxP3^+^ T cells correlated with a good prognosis, whereas other studies found no prognostic association or even a bad prognostic claim [3]–[5], [22]–[31]. The data were organized according to overall survival and recurrence; then combined results strongly demonstrated that high density of tumor-infiltrating FoxP3^+^ T cells was a good prognosis for CRC. The result has challenged the conventionally theory that FoxP3^+^ T cells can suppress tumor immunity. It is regrettable that very few studies in the literature have examined the exact functional properties of FoxP3^+^ T cells isolated from human CRC. In considering CRC grows in a septic microenvironment, researchers recently hypothesized that the favorable prognostic effect of FoxP3^+^ T cells may reflect their ability to preferentially suppress tumor-promoting inflammatory responses to gut microbes and Th17-cell-dependent proinflammatory [4].

For GC, the prognostic significance of tumor-infiltrating FoxP3^+^ T cells for the survival of patients with gastric cancer remains controversial. There are 16 studies involving 1873 patients that compared the survival of HCC according to FoxP3^+^ T cells expression level of the primary tumor met the enrollment criteria. In the 16 studies, studies looking at gastric cancers show a split among poor (*n* = 11), neutral (*n* = 2), and good (*n* = 3) prognostic claims. Base on those studies, the Meta-Analysis results consistent with HCC, high density of FoxP3^+^ T cells was associated with poor survival and high recurrences.

However, one should be cautious when interrupting these results due to the limitations of our studies. Further high-quality studies are still needed to confirm these results. There are several important limitations also need to be considered. First of all, patients had received different treatments and postoperative treatment; preoperative TNM category and histologic types were various. Whereas, we were unable to assess these potential confounders present in individual studies. Second, although we tried to identify all relevant data, potential publication bias was unavoidable and some data could still be missing. Third, the antibody, cell-scoring strategy and the cutoff value were defined differently in some studies. Finally, this study was constrained to studies published in English and Chinese language; it was difficult to completely rule out publication bias.

HCC, CRC and GC are gastrointestinal tumors, and come from immune tolerance organs which exposed to high levels exogenous antigens. However, the role and function of FoxP3^+^ T cells were different completely. Thus, the original view that FoxP3^+^ T cells invariably suppress tumor immunity is oversimplified for CRC. The discrepancy in different tumors could arise from differences in study methodologies or in the biologic properties of specific tumor types. We require better understanding of the functional subtypes of FoxP3^+^ T cells and their biologic properties in different tumor microenvironments if we wish to rationally modulate their behavior to enhance tumor immunity. We believe that the interaction between the different components of the tumor microenvironment and the diversity of signals provided by the tumor cells can explain these discrepancies in the prognostic studies relying on the presence of Tregs in tumor infiltrates. Recent findings have shown that a subset of FoxP3^+^ Tregs could acquire the capacity to produce IL-17 instead of IL-10 and TGF-β [52]. The double-positive T cells exhibit functions of both Th17 and Tregs, or act as a transient population that may eventually generate either Th17 or Tregs, presenting a potential mechanism for the Tregs/Th17 regulation in the progression of tumor.

In summary, some studies fit with the general notion that FoxP3^+^ T cells suppress adaptive immune responses and led many groups to pursue strategies to deplete FoxP3^+^ T cells from patients or mouse with cancer as a means to enhance tumor immunity [53]–[55]. However, our findings suggest that the treatment of depletion or attenuation of FoxP3^+^ T cells can be used for the treatment of HCC and GC but detrimental for CRC.

## Supporting Information

Figure S1 **Flow diagram of study selection.** A: Flow diagram of study selection for HCC; B: Flow diagram of study selection for CRC; C: Flow diagram of study selection for GC.
(DOC)

Figure S2 **Funnel plots for HCC.** A: 12 articles in the meta-analysis of survival during follow-up 1-year after treatment; B: 11 articles in the meta-analysis of survival during follow-up 3-year after treatment; C: 12 articles in the meta-analysis of survival during follow-up 5-year after treatment; D: 11 articles in the meta-analysis of recurrence during follow-up 1-year after treatment; E: 10 articles in the meta-analysis of recurrence during follow-up 3-year after treatment; F: 11 articles in the meta-analysis of recurrence during follow-up 5-year after treatment.
(TIF)

Figure S3 **Funnel plots for CRC.** A: 8 articles in the meta-analysis of survival during follow-up 1-year after treatment; B: 8 articles in the meta-analysis of survival during follow-up 3-year after treatment; C: 8 articles in the meta-analysis of survival during follow-up 5-year after treatment; D: 6 articles in the meta-analysis of recurrence during follow-up 1-year after treatment; E: 6 articles in the meta-analysis of recurrence during follow-up 3-year after treatment; F: 6 articles in the meta-analysis of recurrence during follow-up 5-year after treatment.
(TIF)

Figure S4 **Funnel plots for GC.** A: 16 articles in the meta-analysis of survival during follow-up 1-year after treatment; B: 16 articles in the meta-analysis of survival during follow-up 3-year after treatment; C: 16 articles in the meta-analysis of survival during follow-up 5-year after treatment; D: 4 articles in the meta-analysis of recurrence during follow-up 1-year after treatment; E: 4 articles in the meta-analysis of recurrence during follow-up 3-year after treatment; F: 4 articles in the meta-analysis of recurrence during follow-up 5-year after treatment.
(TIF)

Checklist S1 **PRISMA Checklist.**
(ZIP)

## References

[1] Grivennikov SI, Greten FR, Karin M (2010) Immunity, inflammation, and cancer. Cell 140: 883–899. 20303878 10.1016/j.cell.2010.01.025 PMC2866629

[2] Sakaguchi S, Sakaguchi N, Asano M, Itoh M, Toda M (1995) Immunologic self-tolerance maintained by activated T cells expressing IL-2 receptor α-chains (CD25). Breakdown of a single mechanism of self-tolerance causes various auto-immune diseases. J Immunol 155: 1151–1164. 7636184

[3] deLeeuw RJ, Kost SE, Kakal JA, Nelson BH (2012) The prognostic value of FoxP3+ tumor-infiltrating lymphocytes in cancer: a critical review of the literature. Clin Cancer Res 18: 3022–3029. 22510350 10.1158/1078-0432.CCR-11-3216

[4] Ladoire S, Martin F, Ghiringhelli F (2011) Prognostic role of FOXP3+ regulatory T cells infiltrating human carcinomas: the paradox of colorectal cancer. Cancer Immunol Immunother 60: 909–918. 21644034 10.1007/s00262-011-1046-y PMC11028605

[5] Mei Z, Liu Y, Liu C, Cui A, Liang Z, et al. (2014) Tumour-infiltrating inflammation and prognosis in colorectal cancer: systematic review and meta-analysis. Br J Cancer. doi: 10.1038/bjc.2014.46. 10.1038/bjc.2014.46 PMC3960618 24504370

[6] Tierney JF, Stewart LA, Ghersi D, Burdett S, Sydes MR (2007) Practical methods for incorporating summary time-to-event data into meta-analysis. Trials 8: 16. 17555582 10.1186/1745-6215-8-16 PMC1920534

[7] Liu JL, Gao W, Kang QM, Zhang XJ, Yang SG (2013) Prognostic value of survivin in patients with gastric cancer: a systematic review with meta-analysis. PLoS One 8: e71930. 23936532 10.1371/journal.pone.0071930 PMC3732238

[8] Han K, Qi W, Gan Z, Shen Z, Yao Y, Min D (2013) Prognostic value of Ezrin in solid tumors: a meta-analysis of the literature. PLoS One 8: e68527. 23894313 10.1371/journal.pone.0068527 PMC3716773

[9] Gao Q, Qiu SJ, Fan J, Zhou J, Wang XY, et al (2007) Intratumoral balance of regulatory and cytotoxic T cells is associated with prognosis of hepatocellular carcinoma after resection. J Clin Oncol 25: 2586–2593. 17577038 10.1200/JCO.2006.09.4565

[10] Kobayashi N, Hiraoka N, Yamagami W, Ojima H, Kanai Y, et al (2007) FOXP3+ regulatory T cells affect the development and progression of hepatocarcinogenesis. Clin Cancer Res 13: 902–911. 17289884 10.1158/1078-0432.CCR-06-2363

[11] Sasaki A, Tanaka F, Mimori K, Inoue H, Kai S, et al (2008) Prognostic value of tumor-infiltrating FOXP3 regulatory T cells in patients with hepatocellular carcinoma. Eur J Surg Oncol 34: 173–179. 17928188 10.1016/j.ejso.2007.08.008

[12] Li SP, Peng QQ, Ding T, Xu J, Zhang CQ, et al (2008) Clinical significance of regulatory T cells proportion in the peripheral blood and tumor tissue in primary hepatocellular carcinoma. Zhonghua Zhong Liu Za Zhi 30: 523–527. 19062720

[13] Shen SL, Liang LJ, Peng BG, He Q, Kuang M, et al (2011) Foxp3+ regulatory T cells and the formation of portal vein tumour thrombus in patients with hepatocellular carcinoma. Can J Surg 54: 89–94. 21251418 10.1503/cjs.028009 PMC3116706

[14] Zhou J, Ding T, Pan W, Zhu LY, Li L, et al (2009) Increased intratumoral regulatory T cells are related to intratumoral macrophages and poor prognosis in hepatocellular carcinoma patients. Int J Cancer 125: 1640–1648. 19569243 10.1002/ijc.24556

[15] Lin GH, Wang J, Li SH, Wang J, Xu L, et al (2010) Relationship and clinical significance of TGF-beta1 expression with Treg cell infiltration in hepatocellular carcinoma. Chin J Cancer 29: 403–407. 20346216 10.5732/cjc.009.10628

[16] Chen KJ, Lin SZ, Zhou L, Xie HY, Zhou WH, et al (2011) Selective Recruitment of Regulatory T Cell through CCR6-CCL20 in Hepatocellular Carcinoma Fosters Tumor Progression and Predicts Poor Prognosis. PLoS One 6: e24671. 21935436 10.1371/journal.pone.0024671 PMC3173477

[17] Chen KJ, Zhou L, Xie HY, Ahmed TE, Feng XW, et al (2012) Intratumoral regulatory T cells alone or in combination with cytotoxic T cells predict prognosis of hepatocellular carcinoma after resection. Med Oncol 29: 1817–1826. 21678026 10.1007/s12032-011-0006-x

[18] Huang Y, Wang FM, Wang T, Wang YJ, Zhu ZY, et al (2012) Tumor-infiltrating FoxP3+ Tregs and CD8+ T cells affect the prognosis of hepatocellular carcinoma patients. Digestion 86: 329–337. 23207161 10.1159/000342801

[19] Wu H, Chen P, Liao R, Li YW, Yi Y, et al (2012) Overexpression of Galectin-1 Associates with Poor Prognosis in Human Hepatocellular Carcinoma Following Resection. J Gastroenterol Hepatol 27: 1312–1319. 22432916 10.1111/j.1440-1746.2012.07130.x

[20] Huang Y, Wang F, Wang Y, Zhu Z, Gao Y, et al.. (2013) Intrahepatic IL-17+ T cells and FoxP3+ Tregs cooperate to promote development and affect the prognosis of hepatocellular carcinoma.J Gastroenterol Hepatol. doi: 10.1111/jgh.12418. 10.1111/jgh.12418 24303990

[21] Lin SZ, Chen KJ, Xu ZY, Chen H, Zhou L, et al (2013) Prediction of Recurrence and Survival in Hepatocellular Carcinoma Based on Two Cox Models Mainly Determined by FoxP3 Regulatory T Cells. Cancer Prev Res 6: 594–602. 10.1158/1940-6207.CAPR-12-0379 23599540

[22] Sinicrope FA, Rego RL, Ansell SM, Knutson KL, Foster NR, et al (2009) Intraepithelial effector (CD3+)/regulatory (FoxP3+) T-cell ratio predicts a clinical outcome of human colon carcinoma. Gastroenterology 137: 1270–1279. 19577568 10.1053/j.gastro.2009.06.053 PMC2873775

[23] Lee WS, Park S, Lee WY, Yun SH, Chun HK (2010) Clinical impact of tumor-infiltrating lymphocytes for survival in stage II colon cancer. Cancer 116: 5188–5199. 20665489 10.1002/cncr.25293

[24] Suzuki H, Chikazawa N, Tasaka T, Wada J, Yamasaki A, et al (2010) Intratumoral CD8(+) T/FOXP3 (+) cell ratio is a predictive marker for survival in patients with colorectal cancer. Cancer Immunol Immunother 59: 653–661. 19908042 10.1007/s00262-009-0781-9 PMC11030791

[25] Frey DM, Droeser RA, Viehl CT, Zlobec I, Lugli A, et al (2010) High frequency of tumor-infiltrating FOXP3(+) regulatory T cells predicts improved survival in mismatch repair-proficient colorectal cancer patients. Int J Cancer 126: 2635–2643. 19856313 10.1002/ijc.24989

[26] Nosho K, Baba Y, Tanaka N, Shima K, Hayashi M, et al (2010) Tumour-infiltrating T-cell subsets, molecular changes in colorectal cancer, and prognosis: cohort study and literature review. J Pathol 222: 350–366. 20927778 10.1002/path.2774 PMC3033700

[27] Tosolini M, Kirilovsky A, Mlecnik B, Fredriksen T, Mauger S, et al (2011) Clinical impact of different classes of infiltrating T cytotoxic and helper cells (Th1, th2, treg, th17) in patients with colorectal cancer. Cancer Res 71: 1263–1271. 21303976 10.1158/0008-5472.CAN-10-2907

[28] Yoon HH, Orrock JM, Foster NR, Sargent DJ, Smyrk TC, et al (2012) Prognostic impact of FoxP3+ regulatory T cells in relation to CD8+ T lymphocyte density in human colon carcinomas. PLoS One 7: e42274. 22879926 10.1371/journal.pone.0042274 PMC3412852

[29] Suzuki H, Onishi H, Morisaki T, Tanaka M, Katano M (2013) Intratumoral FOXP3+VEGFR2+ regulatory T cells are predictive markers for recurrence and survival in patients with colorectal cancer. Clin Immunol 146: 26–33. 23202541 10.1016/j.clim.2012.10.007

[30] Zeestraten EC, Van Hoesel AQ, Speetjens FM, Menon AG, Putter H, et al (2013) FoxP3- and CD8-positive Infiltrating Immune Cells Together Determine Clinical Outcome in Colorectal Cancer. Cancer Microenviron 6: 31–39. 21732187 10.1007/s12307-011-0071-x PMC3601218

[31] Kim M, Grimmig T, Grimm M, Lazariotou M, Meier E, et al (2013) Expression of Foxp3 in colorectal cancer but not in Treg cells correlates with disease progression in patients with colorectal cancer. PLoS One 8: e53630. 23382847 10.1371/journal.pone.0053630 PMC3559740

[32] Mizukami Y, Kono K, Kawaguchi Y, Akaike H, Kamimura K, et al (2008) Localisation pattern of Foxp3+ regulatory T cells is associated with clinical behaviour in gastric cancer. Br J Cancer 98: 148–153. 18087278 10.1038/sj.bjc.6604149 PMC2359685

[33] Perrone G, Ruffini PA, Catalano V, Spino C, Santini D, et al (2008) Intratumoural FOXP3-positive regulatory T cells are associated with adverse prognosis in radically resected gastric cancer. Eur J Cancer 44: 1875–1882. 18617393 10.1016/j.ejca.2008.05.017

[34] Haas M, Dimmler A, Hohenberger W, Grabenbauer GG, Niedobitek G, et al (2009) Stromal regulatory T-cells are associated with a favourable prognosis in gastric cancer of the cardia. BMC Gastroenterol 9: 65. 19732435 10.1186/1471-230X-9-65 PMC2749861

[35] Shen Z, Zhou S, Wang Y, Li RL, Zhong C, et al (2010) Higher intratumoral infiltrated Foxp3+ Treg numbers and Foxp3+/CD8+ ratio are associated with adverse prognosis in resectable gastric cancer. J Cancer Res Clin Oncol 136: 1585–1595. 20221835 10.1007/s00432-010-0816-9 PMC11828043

[36] Du L, Xiao X, Wang C, Zhang X, Zheng N, et al (2011) Human leukocyte antigen-G is closely associated with tumor immune escape in gastric cancer by increasing local regulatory T cells. Cancer Sci 102: 1272–1280. 21466615 10.1111/j.1349-7006.2011.01951.x

[37] Kim HI, Kim H, Cho HW, Kim SY, Song KJ, et al (2011) The ratio of intra-tumoral regulatory T cells (Foxp3+)/helper T cells (CD4+) is a prognostic factor and associated with recurrence pattern in gastric cardia cancer. J Surg Oncol 104: 728–733. 21792941 10.1002/jso.22038

[38] Lu X, Liu J, Li H, Li W, Wang X, et al (2011) Conversion of intratumoral regulatory T cells by human gastric cancer cells is dependent on transforming growth factor-β1. J Surg Oncol 104: 571–577. 21695703 10.1002/jso.22005

[39] Shu P, Qin J, Qin XY, Sun YH, Shen ZB, et al (2011) Expression and prognostic value of M2 macrophages and regulatory T cells in gastric carcinoma tissue. Zhonghua Wei Chang Wai Ke Za Zhi 14: 368–371. 21614694

[40] Wang B, Xu D, Yu X, Ding T, Rao H, et al (2011) Association of intra-tumoral infiltrating macrophages and regulatory T cells is an independent prognostic factor in gastric cancer after radical resection. Ann Surg Oncol 18: 2585–2593. 21347781 10.1245/s10434-011-1609-3

[41] Ishigami S, Arigami T, Uenosono Y, Matsumoto M, Okumura H, et al (2012) Cancerous HLA class I expression and regulatory T cell infiltration in gastric cancer. Cancer Immunol Immunother 61: 1663–1669. 22374482 10.1007/s00262-012-1225-5 PMC11028633

[42] Kashimura S, Saze Z, Terashima M, Soeta N, Ohtani S, et al (2012) CD83(+) dendritic cells and Foxp3(+) regulatory T cells in primary lesions and regional lymph nodes are inversely correlated with prognosis of gastric cancer. Gastric Cancer 15: 144–153. 22083420 10.1007/s10120-011-0090-9

[43] Yoshii M, Tanaka H, Ohira M, Muguruma K, Iwauchi T, et al (2012) Expression of Forkhead box P3 in tumour cells causes immunoregulatory function of signet ring cell carcinoma of the stomach. Br J Cancer 106: 1668–1674. 22569001 10.1038/bjc.2012.141 PMC3349176

[44] Deng B, Zhu JM, Wang Y, Liu TT, Ding YB, et al (2013) Intratumor hypoxia promotes immune tolerance by inducing regulatory T cells via TGF-β1 in gastric cancer. PLoS One 8: e63777. 23723999 10.1371/journal.pone.0063777 PMC3664556

[45] Kim KJ, Lee KS, Cho HJ, Kim YH, Yang HK, et al (2013) Prognostic implications of tumor-infiltrating FoxP3+ regulatory T cells and CD8+ cytotoxic T cells in microsatellite-unstable gastric cancers. Hum Pathol 45: 285–293. 24331841 10.1016/j.humpath.2013.09.004

[46] Zhou S, Xu S, Tao H, Zhen Z, Chen G, et al (2013) CCR7 Expression and Intratumoral FOXP3(+) Regulatory T Cells are Correlated with Overall Survival and Lymph Node Metastasis in Gastric Cancer. PLoS One 8: e74430. 24040244 10.1371/journal.pone.0074430 PMC3764061

[47] Ma GF, Miao Q, Liu YM, Gao H, Lian JJ, et al.. (2014) High FoxP3 expression in tumour cells predicts better survival in gastric cancer and its role in tumour microenvironment. Br J Cancer. doi: 10.1038/bjc.2014.47. 10.1038/bjc.2014.47 PMC3960619 24548868

[48] Chang KM (2005) Regulatory T cells and the liver: a new piece of the puzzle. Hepatology 41: 700–702. 15789365 10.1002/hep.20678

[49] Kosmaczewska A, Ciszak L, Potoczek S, Frydecka I (2008) The significance of Treg cells in defective tumor immunity. Arch Immunol Ther Exp 56: 181–191. 10.1007/s00005-008-0018-1 18512029

[50] Sakaguchi S (2005) Naturally arising Foxp3-expressing CD25+CD4+ regulatory T cells in immunological tolerance to self and non-self. Nat Immunol 6: 345–352. 15785760 10.1038/ni1178

[51] Wang F, Jing X, Li G, Wang T, Yang B, Zhu Z, et al (2012) Foxp3+ regulatory T cells are associated with the natural history of chronic hepatitis B and poor prognosis of hepatocellular carcinoma. Liver Int 32: 644–655. 22118340 10.1111/j.1478-3231.2011.02675.x

[52] Kryczek I, Wu K, Zhao E, Wei S, Vatan L, et al (2011) IL-17+ regulatory T cells in the microenvironments of chronic inflammation and cancer. J Immunol 186: 4388–4395. 21357259 10.4049/jimmunol.1003251

[53] Morse MA, Hobeika AC, Osada T, Serra D, Niedzwiecki D, et al (2008) Depletion of human regulatory T cells specifically enhances antigen-specific immune responses to cancer vaccines. Blood 112: 610–618. 18519811 10.1182/blood-2008-01-135319 PMC2481547

[54] Powell DJ Jr, Attia P, Ghetie V, Schindler J, Vitetta ES, et al (2008) Partial reduction of human FOXP3+ CD4 T cells in vivo after CD25-directed recombinant immunotoxin administration. J Immunother 31: 189–198. 18481388 10.1097/CJI.0b013e31815dc0e8 PMC3480218

[55] Zhou S, Tao H, Zhen Z, Chen H, Chen G, et al (2013) Depletion of CD4+ CD25+ regulatory T cells promotes CCL21-mediated antitumor immunity. PLoS One 8: e73952. 24023916 10.1371/journal.pone.0073952 PMC3759453

