# Supplementary material for: Prognostic Value of Tumor-Infiltrating FoxP3+ T Cells in Gastrointestinal Cancers: A Meta Analysis
Source: PLoS One. 2014 May 14;9(5):e94376. doi: 10.1371/journal.pone.0094376 (PMC4020764; doi:10.1371/journal.pone.0094376)
Supplement: Figure S1 — Flow diagram of study selection. A: Flow diagram of study selection for HCC; B: Flow diagram of study selection for CRC; C: Flow diagram of study selection for GC. (DOC) [file pone.0094376.s001.doc]

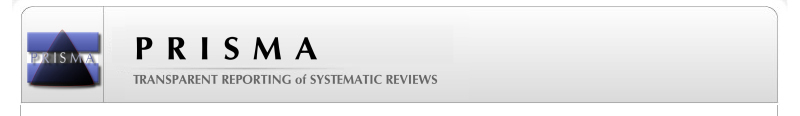
**PRISMA 2009 Flow Diagram**

**Screening**

**Included**

**Eligibility**

**Identification**

Records identified through database searching
(n = 809)

Additional records identified through other sources
(n = 0)

Records after duplicates removed
(n = 393)

Records screened
(n = 393)

Records excluded
(n = 371)

Full-text articles assessed for eligibility
(n = 22)

Full-text articles excluded (n = 9)：Peritumoral specimens (n = 2)
Non-surgical treatment (n = 2)

Patients duplicates (n = 1)

Without detailed data (n = 3) Peripheral Blood specimens (n = 1)

Studies included in qualitative synthesis
(n = 13)

Studies included in quantitative synthesis (meta-analysis)
(n = 13)

**A**

**B**

Records identified through database searching

(n = 863)

**Screening**

**Included**

**Eligibility**

**Identification**

Additional records identified through other sources

(n = 0)

Records after duplicates removed

(n = 237)

Records screened

(n = 237)

Records excluded

(n = 219)

Full-text articles assessed for eligibility

(n = 18)

Full-text articles excluded (n = 8):

Lymph follicles specimens (n = 1)

Without detailed data (n =7)

Studies included in qualitative synthesis

(n = 10)

Studies included in quantitative synthesis (meta-analysis)

(n = 10)

**C**

**Screening**

**Included**

**Eligibility**

**Identification**

Records identified through database searching

(n = 538)

Additional records identified through other sources

(n = 0)

Records after duplicates removed

(n = 194)

Records screened

(n = 194)

Records excluded

(n = 175)

Full-text articles assessed for eligibility

(n = 19)

Full-text articles excluded (n = 3):

Without detailed data (n = 2)

Patients duplicates (n = 1)

Studies included in qualitative synthesis

(n = 16)

Studies included in quantitative synthesis (meta-analysis)

(n = 16)
