# Supplementary figures and images for: Prognostic Value of Tumor-Infiltrating FoxP3+ T Cells in Gastrointestinal Cancers: A Meta Analysis
Source: PLoS One. 2014 May 14;9(5):e94376. doi: 10.1371/journal.pone.0094376 (PMC4020764; doi:10.1371/journal.pone.0094376)

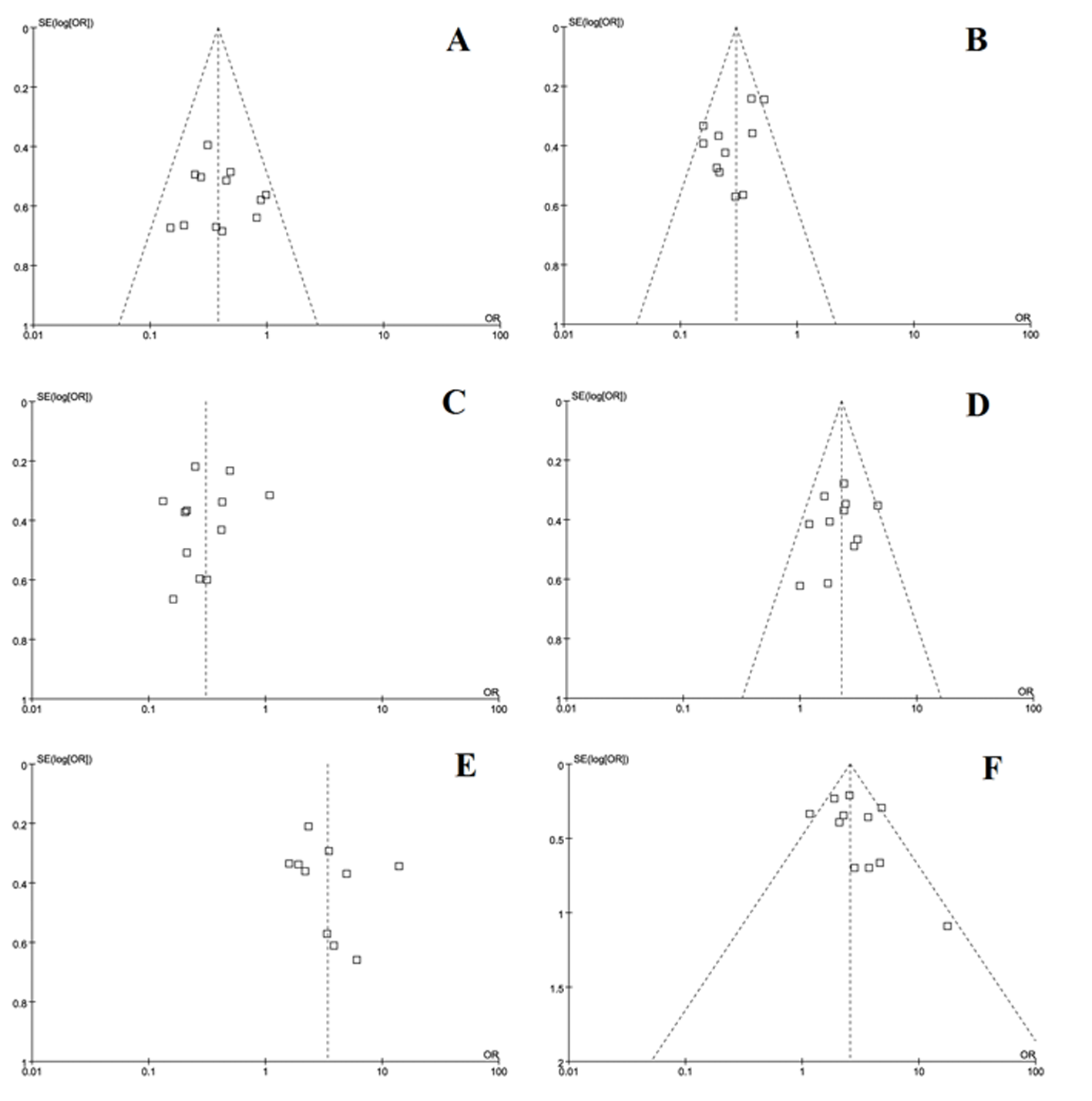

Supplement: Figure S2 — Funnel plots for HCC. A: 12 articles in the meta-analysis of survival during follow-up 1-year after treatment; B: 11 articles in the meta-analysis of survival during follow-up 3-year after treatment; C: 12 articles in the meta-analysis of survival during follow-up 5-year after treatment; D: 11 articles in the meta-analysis of recurrence during follow-up 1-year after treatment; E: 10 articles in the meta-analysis of recurrence during follow-up 3-year after treatment; F: 11 articles in the meta-analysis of recurrence during follow-up 5-year after treatment. (TIF) [file pone.0094376.s002.tif]

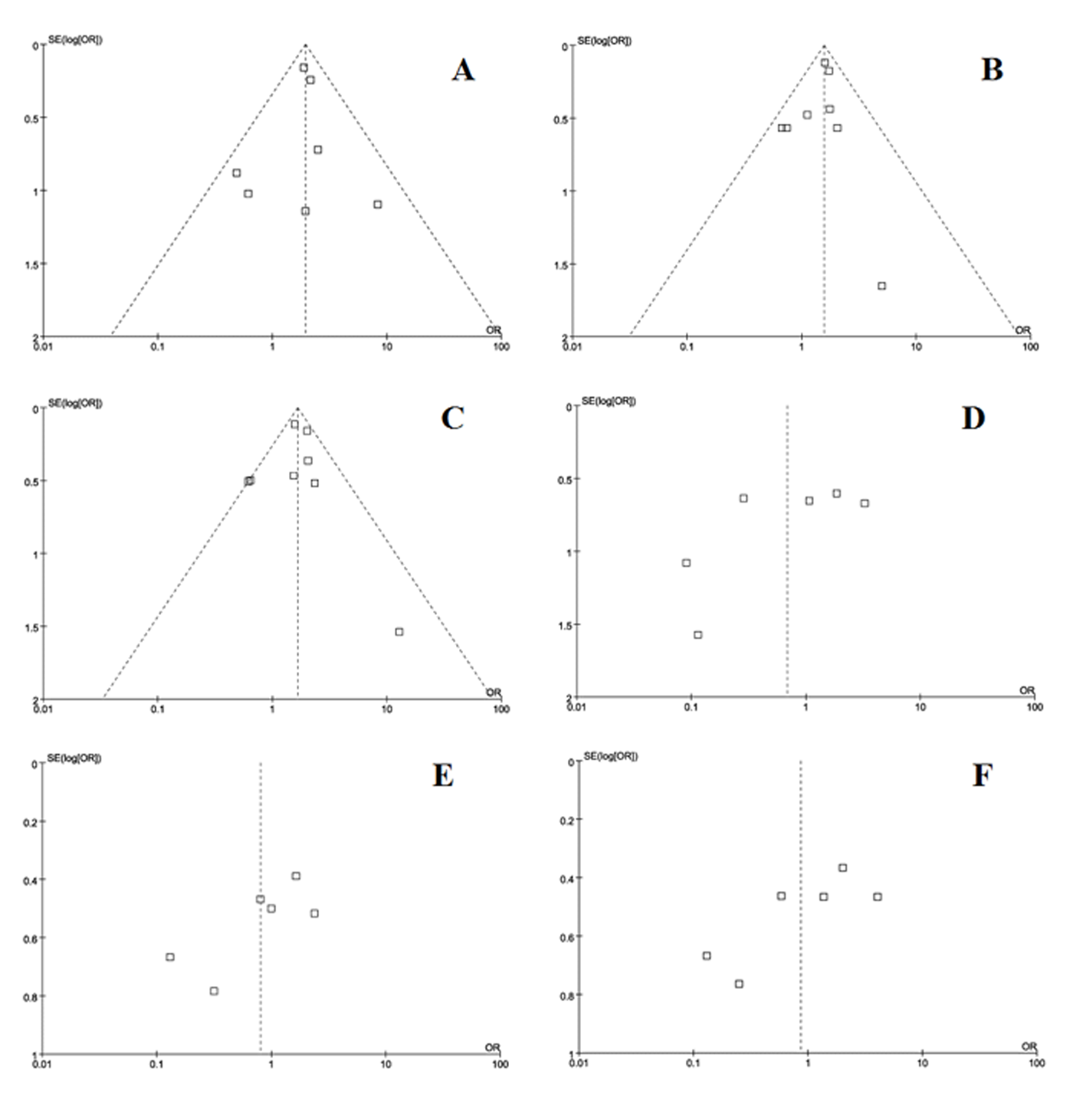

Supplement: Figure S3 — Funnel plots for CRC. A: 8 articles in the meta-analysis of survival during follow-up 1-year after treatment; B: 8 articles in the meta-analysis of survival during follow-up 3-year after treatment; C: 8 articles in the meta-analysis of survival during follow-up 5-year after treatment; D: 6 articles in the meta-analysis of recurrence during follow-up 1-year after treatment; E: 6 articles in the meta-analysis of recurrence during follow-up 3-year after treatment; F: 6 articles in the meta-analysis of recurrence during follow-up 5-year after treatment. (TIF) [file pone.0094376.s003.tif]

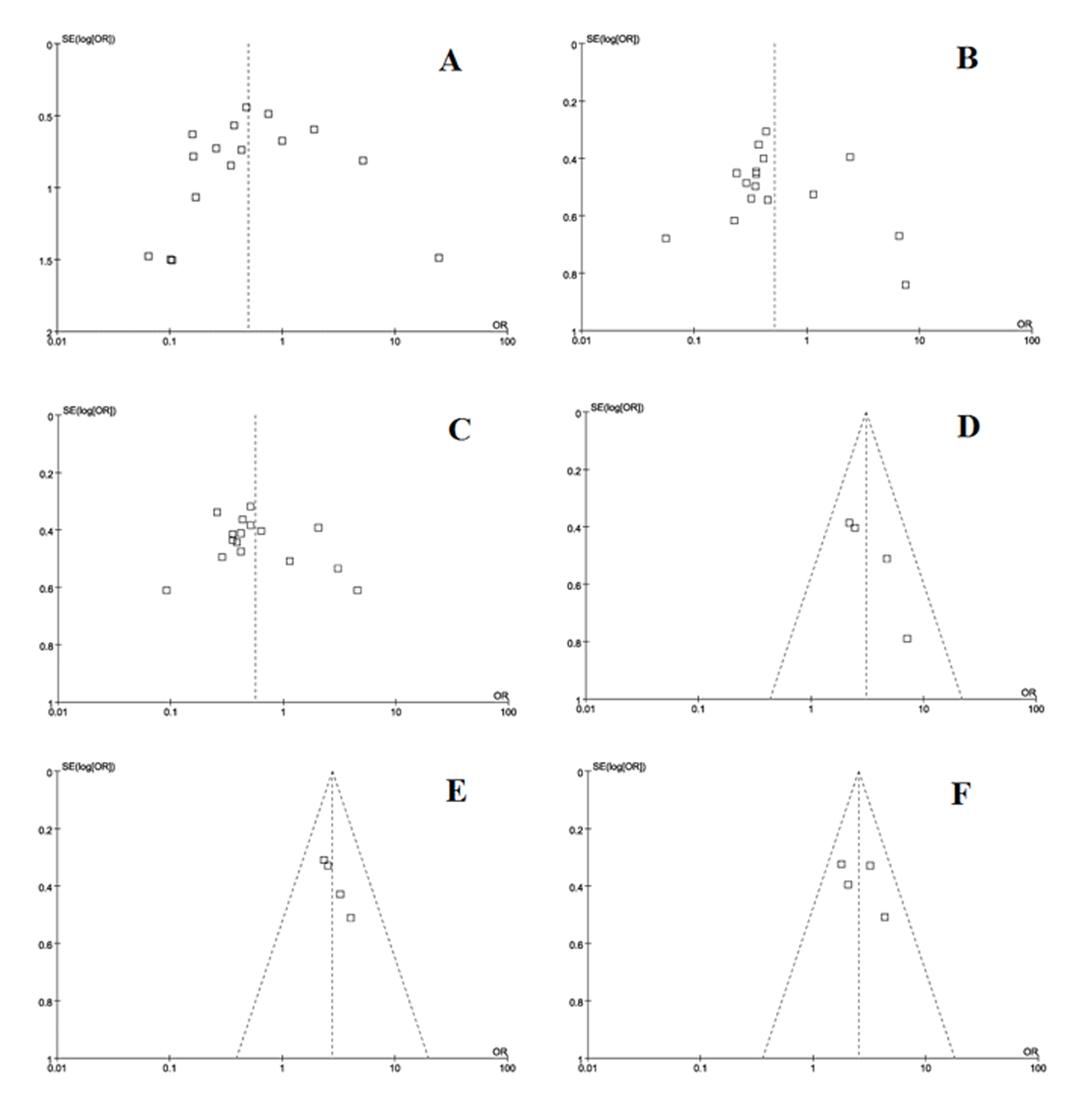

Supplement: Figure S4 — Funnel plots for GC. A: 16 articles in the meta-analysis of survival during follow-up 1-year after treatment; B: 16 articles in the meta-analysis of survival during follow-up 3-year after treatment; C: 16 articles in the meta-analysis of survival during follow-up 5-year after treatment; D: 4 articles in the meta-analysis of recurrence during follow-up 1-year after treatment; E: 4 articles in the meta-analysis of recurrence during follow-up 3-year after treatment; F: 4 articles in the meta-analysis of recurrence during follow-up 5-year after treatment. (TIF) [file pone.0094376.s004.tif]
