# Supplementary material for: Prognostic Value of Tumor-Infiltrating FoxP3+ T Cells in Gastrointestinal Cancers: A Meta Analysis
Source: PLoS One. 2014 May 14;9(5):e94376. doi: 10.1371/journal.pone.0094376 (PMC4020764; doi:10.1371/journal.pone.0094376)
Supplement: Checklist S1 — PRISMA Checklist. (ZIP) [file pone.0094376.s005.zip › ╨┬╜¿╬─╝■╝╨/Checklist.doc]

| **Section/topic** | **#** | **Checklist item** | **Reported on page #** |
| --- | --- | --- | --- |
| **TITLE** | | |  |
| Title | 1 | Prognostic Value of Tumor-infiltrating FoxP3^+^ T Cells in Gastrointestinal Cancers: A Meta Analysis | Title |
| **ABSTRACT** | | |  |
| Structured summary | 2 | ***Purpose:*** Tumor-infiltrating FoxP3^+^ T cells have been reported in various human tumors, which impaired cell-mediated immunity and promoted disease progression. However, its prognostic value for survival in patients with different gastrointestinal cancers [hepatocellular carcinoma (HCC), colorectal cancer (CRC), gastric cancer (GC)] remains controversial.  ***Methods:*** Relevant literature was searched using PubMed, Embase, Cochrane, Ovid Medline and Chinese wanfang databases. A meta-analysis was conducted to estimate pooled survival and recurrence ratios. The odds ratio (OR) and 95% confidence intervals (CI) were calculated employing fixed- or random-effects models depending on the heterogeneity of the included trials.  ***Results:*** For HCC and GC, the overall survival at 1, 3 and 5-year of high FoxP3^+^ T cells infiltration patients were lower than low FoxP3^+^ T cells infiltration patients (*P* < 0.05). The recurrences at 1, 3 and 5-year of high FoxP3^+^ T cells infiltration patients were higher than low FoxP3^+^ T cells infiltration patients (*P* < 0.001). But for CRC, the overall survival at 1, 3 and 5-year of high FoxP3^+^ T cells infiltration patients were higher than low FoxP3^+^ T cells infiltration patients (*P* < 0.001). There were no differences in 1, 3 and 5-year recurrences between high and low FoxP3^+^ T cells infiltration patients (*P* > 0.05).  ***Conclusions:*** Our findings suggested that tumor-infiltrating FoxP3^+^ T cells were a factor for a poor prognosis for HCC and GC, but a good prognosis for CRC. | Abstract |
| **INTRODUCTION** | | |  |
| Rationale | 3 | Abundance tumor-infiltrating FoxP3^+^ T cells are expected to be associated with an unfavorable prognosis, as expected from their capacity to inhibit antitumor immunity. However, this idea has been challenged by recent studies showing that, high tumor infiltration by FoxP3^+^ T cells is not always associated with a poor prognosis. On the contrary, it can improve survival in some tumors [3-5]. It was inconsistent with the initial hypothesis that FoxP3^+^ T cells inhibit antitumor immunity. Even in the same kind of tumor, this conclusion was not entirely consistent [4, 5]. The discrepancy was very obvious, especially in the gastrointestinal cancers such as hepatocellular carcinoma (HCC), colorectal cancer (CRC) and gastric cancer (GC) which all were considered as inflammation-associated cancers since with rich exogenous antigens. | Introduction |
| Objectives | 4 | We sought to conduct a meta-analysis to estimate the prognostic importance of tumor-infiltrating FoxP3^+^ T cells level for overall survival (OS) and disease-free survival (DFS) among patients with HCC, CRC and GC, aiming to gain insights into whether FoxP3^+^ T cells could provide useful guidance in the biological understanding and treatment of solid tumors. | Introduction |
| **METHODS** | | |  |
| Protocol and registration | 5 | Indicate if a review protocol exists, if and where it can be accessed (e.g., Web address), and, if available, provide registration information including registration number. | No |
| Eligibility criteria | 6 | Case-control and cohort studies, published in English only. | Material and methods |
| Information sources | 7 | Relevant articles were identified by two reviewers via an electronic search of PubMed, EMBASE, Cochrane, Ovid Medline and Chinese wanfang databases. And the search time period of the electronic database was from inception to Feb 8th, 2014. | Material and methods |
| Search | 8 | Studies were searched using the search terms of (FoxP3 or regulatory T cells), (hepatocellular carcinoma, colorectal cancer or gastric cancer) and “prognosis”. | Material and methods |
| Study selection | 9 | Inclusion criteria for this study were as follows: (1) patients were diagnosed clearly; (2) report of FoxP3^+^ T cells in tumor surgical specimens; (3) FoxP3^+^ T cells evaluation using immunohistochemical method; (4) association of high and low FoxP3^+^ T cells infiltration patients with overall survival (OS), and/or disease-free survival (DFS) and contained survival curves. (5) when the same author or group reported results obtained from the same patient population in more than one article, the most recent report or the most informative report was included.  Exclusion criteria for this study were as follows: (1) letters, reviews, case reports, conference abstracts, editorials, and expert opinion were excluded; (2) articles in which have no information on survival rates or survival curve; (3) Non-surgical treatment study; (4) non-primary cancer, such as metastatic cancer or recurrent cancer; (5) peripheral blood or peritumoral specimens. | Material and methods |
| Data collection process | 10 | Data extraction was conducted independently by 2 investigators. Disagreement on article inclusion between the two reviewers was resolved via a third reviewer. | Material and methods |
| Data items | 11 | Author name, publication time, Journal, sex, sample size, marker, antibody, cutoff and survival. | Material and methods |
| Risk of bias in individual studies | 12 | We used funnel plots to assess the publication bias, and tested for funnel plot asymmetry using Egger’s test and Begg’s test. | Material and methods |
| Summary measures | 13 | Odds Ratio, Hazard Ratio | Material and methods |
| Synthesis of results | 14 | All analyses were performed with Review Manager version 5 (RevMan, Cochrane Collaboration, Oxford, England). Statistical heterogeneity between trials was evaluated by χ^2^ test and was considered significant when *P* < 0.05. In the absence of statistically significant heterogeneity, the Mantel-Haenszel method in the fixed-effect model was used for the Meta analysis. Otherwise, the DerSimonian and Laird method in the random-effect model was selected. The odds ratio (OR) with 95%CI was used to assess treatment efficacy. The combined result was an average OR and 95%CI weighted according to the standard error of the OR of the trial. *P* < 0.05 was considered statistically significant. | Material and methods |

Page 1 of 2

| **Section/topic** | **#** | **Checklist item** | **Reported on page #** |
| --- | --- | --- | --- |
| Risk of bias across studies | 15 | The potential publication bias was evaluated by funnel plots. | Material and methods |
| Additional analyses | 16 | Describe methods of additional analyses (e.g., sensitivity or subgroup analyses, meta-regression), if done, indicating which were pre-specified. | No |
| **RESULTS** | | |  |
| Study selection | 17 | See Fig S1. | Results |
| Study characteristics | 18 | See table 1, table2 and table 3. | Results |
| Risk of bias within studies | 19 | See Fig S2, Fig S2 and Fig S3. | Results |
| Results of individual studies | 20 | For HCC and GC, the overall survival at 1, 3 and 5-year of high FoxP3^+^ T cells infiltration patients were lower than low FoxP3^+^ T cells infiltration patients (*P* < 0.05). The recurrences at 1, 3 and 5-year of high FoxP3^+^ T cells infiltration patients were higher than low FoxP3^+^ T cells infiltration patients (*P* < 0.001). But for CRC, the overall survival at 1, 3 and 5-year of high FoxP3^+^ T cells infiltration patients were higher than low FoxP3^+^ T cells infiltration patients (*P* < 0.001). There were no differences in 1, 3 and 5-year recurrences between high and low FoxP3^+^ T cells infiltration patients (*P* > 0.05). | Results |
| Synthesis of results | 21 | **Meta-analysis for HCC**  Survival during follow-up 1, 3, 5-year after surgical resection: The overall survival rate during follow-up 1-year was significantly lower in high FoxP3^+^ T cells infiltration patients (82.8%) than low FoxP3^+^ T cells infiltration patients (92.1%) with a combined OR of 0.38 (95%CI = 0.28-0.52, *P* < 0.001. Figure 1A). The overall survival rate during follow-up 3-year was significantly lower in high FoxP3^+^ T cells infiltration patients (51.8%) than low FoxP3^+^ T cells infiltration patients (76.8%) with a combined OR of 0.30 (95%CI = 0.24-0.37, *P* < 0.001. Figure 1B). The overall survival rate during follow-up 5-year was significantly lower in high FoxP3^+^ T cells infiltration patients (38.4%) than low FoxP3^+^ T cells infiltration patients (64.1%) with a combined OR of 0.31 (95%CI = 0.21-0.44, *P* < 0.001. Figure 1C).  Recurrence during follow-up 1, 3, 5-year after surgical resection: The recurrence rate during follow-up 1-year was significantly higher in high FoxP3^+^ T cells infiltration patients (32.9%) than low FoxP3^+^ T cells infiltration patients (19.0%) with a combined OR of 2.25 (95%CI = 1.79-2.83, *P* < 0.001. Figure 2A). The recurrence rate during follow-up 3-year was significantly higher in high FoxP3^+^ T cells infiltration patients (60.2%) than low FoxP3^+^ T cells infiltration patients (33.8%) with a combined OR of 3.39 (95%CI = 2.22-5.17, *P* < 0.001. Figure 2B). The recurrence rate during follow-up 5-year was significantly higher in high FoxP3^+^ T cells infiltration patients (69.6%) than low FoxP3^+^ T cells infiltration patients (49.6%) with a combined OR of 2.56 (95%CI = 2.09-3.13, *P* < 0.001. Figure 2C).  **Meta-analysis for CRC**  Survival during follow-up 1, 3, 5-year after surgical resection: The overall survival rate during follow-up 1-year was significantly higher in high FoxP3^+^ T cells infiltration patients (91.2%) than low FoxP3^+^ T cells infiltration patients (84.5%) with a combined OR of 1.93 (95%CI = 1.51-2.48, *P* < 0.001. Figure 3A). The overall survival rate during follow-up 3-year was significantly higher in high FoxP3^+^ T cells infiltration patients (76.4%) than low FoxP3^+^ T cells infiltration patients (67.9%) with a combined OR of 1.56 (95%CI = 1.31-1.87, *P* < 0.001. Figure 3B). The overall survival rate during follow-up 5-year was significantly higher in high FoxP3^+^ T cells infiltration patients (69.9%) than low FoxP3^+^ T cells infiltration patients (58.9%) with a combined OR of 1.65 (95%CI = 1.40-1.95, *P* < 0.001. Figure 3C).  Recurrence during follow-up 1, 3, 5-year after surgical resection: There were no differences in 1(OR = 0.69, 95%CI = 0.23-2.01, *P* = 0.49. Figure 4A), 3 (OR=0.80, 95%CI = 0.37-1.72, *P* = 0.57. Figure 4B) and 5-year (OR=0.86, 95%CI = 0.34-2.18, *P* = 0.75. Figure 4C) recurrences between high and low FoxP3^+^ T cells infiltration patients.  **Meta-analysis for GC**  Survival during follow-up 1, 3, 5-year after surgical resection: The overall survival rate during follow-up 1-year was significantly lower in high FoxP3^+^ T cells infiltration patients (87.2%) than low FoxP3^+^ T cells infiltration patients (92.8%) with a combined OR of 0.50 (95%CI = 0.28-0.88, *P* = 0.02. Figure 5A). The overall survival rate during follow-up 3-year was significantly lower in high FoxP3^+^ T cells infiltration patients (65.4%) than low FoxP3^+^ T cells infiltration patients (78.2%) with a combined OR of 0.51 (95%CI = 0.32-0.83, *P* = 0.007. Figure 5B). The overall survival rate during follow-up 5-year was significantly lower in high FoxP3^+^ T cells infiltration patients (55.6%) than low FoxP3^+^ T cells infiltration patients (69.0%) with a combined OR of 0.56 (95%CI = 0.38-0.84, *P* = 0.005. Figure 5C).  Recurrence during follow-up 1, 3, 5-year after surgical resection: The recurrence rate during follow-up 1-year was significantly higher in high FoxP3^+^ T cells infiltration patients (26.9%) than low FoxP3^+^ T cells infiltration patients (10.8%) with a combined OR of 3.06 (95%CI = 1.95-4.80, *P* < 0.001. Figure 6A). The recurrence rate during follow-up 3-year was significantly higher in high FoxP3^+^ T cells infiltration patients (43.4%) than low FoxP3^+^ T cells infiltration patients (22.4%) with a combined OR of 2.77 (95%CI = 1.92-3.98, *P* < 0.001. Figure 6B). The recurrence rate during follow-up 5-year was significantly higher in high FoxP3^+^ T cells infiltration patients (52.5%) than low FoxP3^+^ T cells infiltration patients (33.6%) with a combined OR of 2.52 (95%CI = 1.76-3.62, *P* < 0.001. Figure 6C). | Results |
| Risk of bias across studies | 22 | Publication bias may exist when no significant findings remain unpublished, thus artificially inflating the apparent magnitude of an effect. Survival and recurrences following high and low FoxP3^+^ T cells infiltration patients with HCC, CRC and GC were calculated by the fixed-effect model and random-effect model, respectively. The results were similar and the combined results were highly reliable.  The funnel plots on survival and recurrence following high and low FoxP3^+^ T cells infiltration patients with HCC (Figure S2), CRC (Figure S3) and GC (Figure S4) showed basic symmetry, which suggested no publication bias. | Results |
| Additional analysis | 23 | Give results of additional analyses, if done (e.g., sensitivity or subgroup analyses, meta-regression [see Item 16]). | No |
| **DISCUSSION** | | |  |
| Summary of evidence | 24 | HCC, CRC and GC are gastrointestinal tumors, and come from immune tolerance organs which exposed to high levels exogenous antigens. However, the role and function of FoxP3^+^ T cells were different completely. Thus, the original view that FoxP3^+^ T cells invariably suppress tumor immunity is oversimplified for CRC. The discrepancy in different tumors could arise from differences in study methodologies or in the biologic properties of specific tumor types. We require better understanding of the functional subtypes of FoxP3^+^ T cells and their biologic properties in different tumor microenvironments if we wish to rationally modulate their behavior to enhance tumor immunity. | Discussion |
| Limitations | 25 | Further high-quality studies are still needed to confirm these results. There are several important limitations also need to be considered. First of all, patients had received different treatments and postoperative treatment; preoperative TNM category and histologic types were various. Whereas, we were unable to assess these potential confounders present in individual studies. Second, although we tried to identify all relevant data, potential publication bias was unavoidable and some data could still be missing. Third, the antibody, cell-scoring strategy and the cutoff value were defined differently in some studies. Finally, this study was constrained to studies published in English and Chinese language; it was difficult to completely rule out publication bias. | Discussion |
| Conclusions | 26 | our findings suggest that the treatment of depletion or attenuation of FoxP3^+^ T cells can be used for the treatment of HCC and GC but detrimental for CRC.. | Discussion |
| **FUNDING** | | |  |
| Funding | 27 | Describe sources of funding for the systematic review and other support (e.g., supply of data); role of funders for the systematic review. | No |

*From:*  Moher D, Liberati A, Tetzlaff J, Altman DG, The PRISMA Group (2009). Preferred Reporting Items for Systematic Reviews and Meta-Analyses: The PRISMA Statement. PLoS Med 6(6): e1000097. doi:10.1371/journal.pmed1000097

For more information, visit: **www.prisma-statement.org**.

Page 2 of 2
